# The Diagnostic Value of Copy Number Variants in Genetic Cardiomyopathies and Channelopathies

**DOI:** 10.3390/jcdd12070258

**Published:** 2025-07-04

**Authors:** Valerio Caputo, Virginia Veronica Visconti, Enrica Marchionni, Valentina Ferradini, Clara Balsano, Pasquale De Vico, Leonardo Calò, Ruggiero Mango, Giuseppe Novelli, Federica Sangiuolo

**Affiliations:** 1 Department of Life, Health and Environmental Sciences, University of L’Aquila, 67100 L’Aquila, Italy; valerio.caputo@univaq.it (V.C.); clara.balsano@univaq.it (C.B.); 2 Fondazione Francesco Balsano, 00198 Rome, Italy; novelli@med.uniroma2.it; 3 Department of Biomedicine and Prevention, University of Rome Tor Vergata, 00133 Rome, Italy; virginia.veronica.visconti@uniroma2.it (V.V.V.); ferradini@med.uniroma2.it (V.F.); 4 Medical Genetics Unit, Tor Vergata University Hospital, 00133 Rome, Italy; enrica.marchionni@ptvonline.it; 5 Department of Anaesthesia, University of Rome Tor Vergata, 00133 Rome, Italy; pasquale.devico@ptvonline.it; 6 Department of Cardiology, Policlinico Casilino, 00169 Rome, Italy; leonardocalo.doc@gmail.com; 7 Cardiology Unit, Department of Emergency and Critical Care, Policlinico Tor Vergata, 00133 Rome, Italy; mango@med.uniroma2.it

**Keywords:** cardiomyopathies (CMPs), dilated cardiomyopathy (DCM), hypertrophic cardiomyopathy (HCM), arrhythmogenic cardiomyopathy (ACM), channelopathies (CNPs), long QT syndrome (LQTS), Brugada syndrome (BrS), catecholaminergic polymorphic ventricular tachycardia (CPVT), copy number variants (CNVs), genetic testing

## Abstract

Sudden cardiac death represents an unexpected death for which a strong underlying genetic background has been described. The primary causes are identified in cardiomyopathies and channelopathies, which are heart diseases of the muscle and electrical system, respectively, without coronary artery disease, hypertension, valvular disease, and congenital heart malformations. Genetic variants, especially single nucleotide variants and short insertions/deletions impacting essential myocardial functions, have shown that cardiomyopathies display high heritability. However, genetic heterogeneity, incomplete penetrance, and variable expression may complicate the interpretation of genetic findings, thus delaying the management of seriously at-risk patients. Moreover, recent studies show that the diagnostic yield related to genetic cardiomyopathies ranges from 28 to 40%, raising the need for further research. In this regard, investigating the occurrence of structural variants, especially copy number variants, may be crucial. Based on these considerations, this review aims to provide an overview of copy number variants identified in cardiomyopathies and discuss them, considering diagnostic yield. This review will ultimately address the necessity of incorporating copy number variants into routine genetic testing for cardiomyopathies and channelopathies, a process increasingly enabled by advances in next-generation sequencing technologies.

## 1. Introduction

Sudden cardiac death (SCD) is an unexpected, sudden death due to cardiac causes within one hour of symptom onset [1].

The most of SCD-related diseases are characterized by an underlying genetic component, which allows these congenital heart conditions to be broadly grouped into two categories: cardiomyopathies (CMPs) and channelopathies (CNPs) [2].

CMPs include a heterogeneous group of structural and functional abnormalities of the myocardium, including Hypertrophic Cardiomyopathy (HCM; MIM #192600), Dilated Cardiomyopathy (DCM; MIM #115200), Left Ventricular Non-Compaction (LVNC; MIM #604169), and Arrhythmogenic Cardiomyopathy (ACM; MIM #107970) [3].

Otherwise, CNPs are primarily electrical disorders affecting ion channels, including Brugada Syndrome (BrS; MIM #601144), Long-QT Syndrome (LQTS; MIM #192500), Short-QT Syndrome (SQTS; MIM #609620), and catecholaminergic polymorphic ventricular tachycardia (CPVT; #604772) [4]. Identifying patients at risk of arrhythmic events is challenging, as SCD itself may be the first symptom of such conditions [5]. This issue can be prevented by performing genetic testing, which can establish a specific diagnosis, improving the prognostic insight of the individual and at-risk family members. Broader testing platforms, including whole-exome (WES) and whole-genome sequencing (WGS), are becoming increasingly available and affordable and may play a larger role in clinical care and biomedical investigation [6,7]. These tests can identify novel disease-associated genes and uncover new insights into pathogenic mechanisms and causal pathways, especially when integrated with a thorough phenotypic assessment of probands and their relatives.

The diagnostic yield of genetic CMPs and CNPs seems to range from 28 to 40%, raising the need for further research [8].

In this context, Copy Number Variants (CNVs), a subset of structural variants (SVs), have recently been recognized as a major contributor to genomic structural variation, accounting for over 10% of human genetic inheritance [9,10]. This type of genetic variability involves both duplications and deletions, especially those ranging from 1000 base pairs to 5 megabases. It is often underestimated because current routinary diagnostic tests mainly aim at identifying single nucleotide variants (SNVs) and short insertions/deletions (indels) [11,12]. Therefore, assessing the role of CNVs in the pathogenesis of CMPs and CNPs is a fruitful strategy to improve their diagnosis.

This review aims to provide an overview of the current literature data describing the contribution of CNVs in genetic CMPs, including DCM, ACM, HCM, and CNPs, namely LQTs, Brs, and CPVT. The ultimate goal is to highlight the importance of integrating CNV analysis within the routinely diagnostic workflow, with the aim of providing a genetic diagnosis for improving the clinical management of patients and their families.

## 2. Structural Cardiomyopathies

Structural CMPs are characterized by alteration of the heart at structural and functional levels, as mentioned above. Overall, such structural alterations are mainly due to genetic variants affecting sarcomeres components, desmosomes, cytoskeleton, and the nuclear envelope [13]. Interestingly, besides having identified several causative SNVs, also various CNVs have been detected in DCM, ACM, and HCM patients, providing useful insights into their role in the pathogenesis (Table 1).

### 2.1. Dilated Cardiomyopathy

DCM is a progressive disease of heart muscle characterized by left ventricular (LV) enlargement and systolic dysfunction, occurring in the absence of abnormal loading conditions or coronary artery disease [27]. Familial DCM is associated with rare variant mutations in more than 30 genes, mostly encoding sarcomere or sarcomere-associated proteins [28]. To date, approximately 65% of DCM cases test negative for SNVs through routine genetic screening, highlighting the need to investigate other genetic variants.

To explore the potential role of CNVs in DCM, Norton et al. analyzed 311 probands with DCM, performing genome-wide copy number analysis. Their analysis revealed a 8733 bp deletion, encompassing exon 4 of the heat shock protein cochaperone *BAG cochaperone 3* (*BAG3*; OMIM *603883), segregating in seven DCM-affected family members. The mechanism responsible for the clinical phenotype is still uncertain, but it is speculated that *BAG3* deletion probably results in loss of function or protein haploinsufficiency [22]. The same research group also performed a multiplex ligation-dependent probe amplification (MLPA) approach to detect possible deletions and duplications in *Lamin A/C* (*LMNA*; OMIM *150330), one of the most frequently mutated genes in DCM. However, no CNVs were identified among 58 DCM patients previously tested negative for SNVs in *LMNA* and other known DCM-associated genes [29].

In contrast, a previous investigation in a smaller cohort of 25 DCM patients reported a large deletion within *LMNA* encompassing exons 3 to 12 in a patient with major nuclear envelope abnormalities, suggesting haploinsufficiency as a potential pathogenic mechanism [23].

More recently, renewed efforts have been made to reanalyze cases previously negative for DCM genetic testing. A study involving 1504 patients with clinically suspected DCM selected from a larger cohort employed a secondary next-generation sequencing (NGS)-based CNV analysis using a bioinformatics pipeline that includes a CNVkit and in-house technology [24]. This analysis identified nine multigene and intragenic CNVs associated with DCM phenotype in different patients: a large multigene deletion of 13.7 Mb, the loss of *GATA binding protein 4* (*GATA4*; OMIM *600576) sequence due to a 3.7 Mb deletion and a 51.9 Kb deletion within *T-box transcription factor 20* (*TBX20*; OMIM *606061). In *DMD* gene a deletion encompassing exons 48–51 and a duplication encompassing exons 19–37 was evidenced. Interestingly, CNVs within genes strongly known as associated with DCM were also detected, including a 3 kb del/ins of *LMNA*, a deletion of *Plakophilin 2* (*PKP2*; OMIM *602861) exon 8 and a deletion of *Desmoplakin* (*DSP*; OMIM *125647) exon 1 [24]. Further studies underscore the relevance of large deletions occurring in early-onset DCM-associated genes, such as the one described in the *RNA binding motif protein 20* (*RBM20*; OMIM *613171) gene. A 10q25.2 de novo deletion was identified in a patient who presented with decompensated heart failure at age 16, again suggesting a loss-of-function effect [25]. It is critical to assess the contribution of de novo deletions, such as the one in *Titin* (*TTN*; OMIM *188840) gene that was identified in a boy with DCM at birth [26]. The 86 kb deletion is classified pathogenic using the practice guidelines of American College of Medical Genetics and Genomics (ACMG) and the variant curation criteria of ClinGen consortium related to deletion burden in affected cases and de novo condition. Moreover, it was predicted to remove part of the A-band domain, possibly causing *TTN* loss of function [26,30,31]. Notably, the patient had previously received a negative genetic testing, underscoring the diagnostic utility of CNV analysis [26].

Current evidence indicates that large genomic rearrangements represent a non-neglectable percentage of cases. On this subject, a recent study reported a CNV detection rate of 4.4% (6/136 patients) including deletions and duplications in key DCM genes, such as *DSP* deletion spanning exon 21–23, and *Dystrophin* (*DMD*; OMIM *300377) duplication, spanning exons 45–62 [32]. *DMD* seems to be the gene most frequently identified in association with these large deletions in DCM, as also reported by Uña-Iglesias et al., who determined that 62% of the associated CNVs involving the *DMD* gene [17].

Despite these findings, the contribution of CNVs to DCM pathogenesis remains underexplored, with few large-scale studies to date. These preliminary but compelling results highlight the importance of incorporating CNVs analysis into routine genetic testing for CMPs, which could significantly enhance the detection of the genetic basis of DCM.

### 2.2. Hypertrophic Cardiomyopathy

HCM is an inherited autosomal dominant disease characterized by left ventricular hypertrophy. Although it follows a Mendelian dominant inheritance pattern and numerous causative SNVs have been identified, substantial variability in age of onset, clinical severity, and prognosis is frequently observed even among members of the same family, complicating the clinical diagnosis. Notably, investigations into the role of CNVs in HCM have yielded significant insights, particularly through identifying pathogenic deletions. In particular, a 25 bp deletion within the intron 32 of *Myosin Binding Protein C3* (*MYBPC3*; OMIM *600958) gene was detected in two unrelated Indian families. Functional analyses demonstrated that this deletion affected splicing regulation, ultimately resulting in *MYBPC3* loss of function. However, the variant was also detected in healthy individuals from the same geographic region, suggesting either low penetrance or, more likely, a modifier effect, as proposed by the authors. Consistently, one individual harboring both this deletion and a variant in *Myosin heavy chain 7* (*MYH7*; OMIM *160760) exhibited more severe clinical symptoms than relatives carrying only the *MYBPC3* deletion [14].

In another study, 100 HCM patients who tested negative for causative point variants underwent MLPA analysis targeting CNVs within MYBPC3, which is known to frequently harbor variants introducing premature termination codons (PTCs) and thus causing a loss of protein function. A large 3505 bp deletion (g.47309385_47312889del, c.2905 + 280_*485del), resulting in the loss of exons 27–35 and hypothesized to induce a PTC, was identified in one patient. Breakpoint analysis via long-range PCR suggested that Alu-mediated homologous recombination may underlie this CNV. Notably, the deletion appeared to be extremely rare, and no other CNVs were detected in the remaining cohort [15]. Remarkably, a subsequent independent study using MLPA reported the same deletion in one out of 72 HCM patients who had previously tested negative for pathogenic variants, reinforcing its pathogenic potential [16].

These findings underscore the value of CNV screening in HCM and highlight the importance of data sharing in refining variant classification and interpretation.

A recent large-scale study analyzed by NGS panel-based testing over 11,000 individuals of European ancestry, including 6799 patients clinically diagnosed with HCM. CNVs were confirmed via MLPA and SNP array analyses. This investigation identified 20 distinct likely pathogenic or pathogenic CNVs or short indels, including eleven in *MYBPC3*, one in MYH7, and others in genes such as *PKP2* (*n* = 2), *FHOD3* (*n* = 3; OMIM *609691), *LAMP2* (*n* = 1; OMIM *309060), *PLN* (*n* = 1; OMIM *172405), and *FHL1* (*n* = 1; OMIM *300163). These findings correspond to an overall diagnostic yield of 0.9% for HCM [17].

Interestingly, some CNVs were identified in genes not yet firmly linked to HCM phenotypes, such as *PKP2*, emphasizing the necessity of detailed phenotypic evaluation when interpreting genetic data.

A more comprehensive approach employing WES was applied to a cohort of 14 clinically diagnosed HCM patients, with particular emphasis on large CNV detection. They identified CNVs, mainly deletions, within chromosome 1. More interestingly, they reported five regions harboring common CNVs in every tested patient, although encompassing genes not previously known as involved in HCM. The authors performed bioinformatic analysis to explore the function of these genes, although a clear connection with HCM and cardiac phenotypes needs to be drawn [33].

In contrast, another study, involving 175 individuals with hereditary CMPs, employed MLPA assay in cases with negative WES results. No CNVs were identified, even if MLPA screening was limited to *MYBPC3* and *MYH7* genes only [21].

While caution is warranted in interpreting these findings, especially given the variability in study designs and methodologies, the integration of WES and CNV analyses represents a promising dual approach. This strategy may prove valuable in uncovering additional pathogenic mechanisms in HCM, particularly in patients who are negative on routine genetic testing.

### 2.3. Arrhythmogenic Cardiomyopathy

ACM is associated with serious clinical outcomes, including malignant ventricular arrhythmias, heart failure, and SCD. Despite knowledge on genes associated with these conditions and causative variants identified, it has been estimated that detrimental variants can be identified in about 60% of patients with ACM diagnosis, meaning that for almost 40% of cases the genetic etiology remains unknown [13]. Thus, several efforts have been directed at further elucidating the genetic basis of ACM, particularly through the application of MLPA to detect CNVs. As a result, deletions within ACM genes have been identified.

Roberts et al. investigated CNVs in *PKP2*, a gene commonly implicated in ACM, identifying pathogenic deletions in two affected individuals, previously negative for standard genetic testing.

Notably, one patient was found through MLPA and subsequent array comparative genomic hybridization (CGH array) to harbor a 7.9 Mb deletion at 12p12.1–p11.1, encompassing the entire *PKP2* locus. Another patient displayed a distinct deletion affecting *PKP2*, sparing only exon 1 [18].

A study aimed at creating a Latvian arrhythmogenic right ventricular dysplasia/cardiomyopathy (ARVD-C) registry analyzed 38 patients and their relatives for CNVs in *PKP2* and *DSG2* using MLPA, but it did not identify any deletions or duplications [34].

Conversely, a 2017 Italian study re-evaluated 160 ACM patients who were previously negative for likely pathogenic/pathogenic SNVs in desmosomal genes (namely *PKP2*; *Desmoplakin DSP*, OMIM *125647; *Desmoglein 2 DSG2*, OMIM *125671; *Desmocollin 2 DSC2*, OMIM *125645; *Junction Plakoglobin JUP*, OMIM *173325) and reported an additional diagnostic yield of 7% thanks to CNV analysis. In particular, eleven patients harbored nine distinct CNVs: five had complete deletions of *PKP2*, while others had exon-specific CNVs, including a deletion of exon 4, a multi-exon deletion involving exons 6–11 and a duplication spanning the 5′UTR and exon 1. Additional detected rearrangements included a *DSC2* exon 7–9 duplication and a 482 Kb deletion affecting both *DSC2* and *DSG2* [19].

Further supporting these findings, another study analyzed CNVs via WES combined with confirmatory methods (CGH array, MLPA, and qRT-PCR targeting *PKP2*) in 22 genetically unsolved ACM patients, with MLPA also extended to 50 additional patients. In this cohort of 72 individuals, four *PKP2* deletions were identified (5.6% yield): one spanning exons 1–14, another exons 2–14, one limited to exon 4, and one affecting exons 1–7 [20].

A similar approach was conducted on a large Italian cohort, including 175 patients affected by genetic CMPs. In particular, 29 ACM patients were evaluated by WES and MLPA focusing on *PKP2*, *DSG2*, *DSC2*, *JUP*, *DSP*, *Transforming Growth Factor Beta 3* (*TGFB3*; OMIM *190230), and *Ryanodine Receptor 2* (*RYR2*; OMIM *180902). Three different deletions within *PKP2* were detected by WES and confirmed by MLPA in unrelated patients, namely a deletion of exon 12 and two larger deletions of 72.4 Kb and 75 Kb, respectively, both involving exons 6–14 [21].

The previously illustrated large-scale study analyzing over 11,000 cardiomyopathy patients of European ancestry, including 902 ACM cases, used an NGS panel combined with MLPA and SNP arrays to confirm CNVs. This investigation identified 12 likely pathogenic CNVs in ACM patients: twelve in *PKP2* (including two duplications), two in *DMD* (one duplication), two in *DSP*, and one in *FLNC* (OMIM *102565), achieving a diagnostic yield of 5.8% [17]. This yield closely parallels the findings reported by Fedida et al. [20].

Altogether these studies underscore the predominance of *PKP2* deletions among CNVs associated with ACM. Nevertheless, when the analysis is expanded to include additional ACM-related genes, further CNVs are identified.

## 3. Channelopathies

Inherited CNPs represent a heterogeneous group of genetic disorders, characterized by potentially lethal arrhythmias in structurally normal hearts, accounting for approximately 10–20% of all SCDs [35,36]. Primary electrical heart diseases are caused by genetic mutations in ion channels and mostly include BrS, LQTs, and CPVT [37]. As in inherited CMPs, genetic testing for CNPs primarily relies on NGS to detect SNVs in key genes such as *Potassium voltage-gated channel subfamily Q member 1* (*KCNQ1*; OMIM *607542), *Potassium voltage-gated channel subfamily H member 2* (*KCNH2*; OMIM *152427), and *Sodium voltage-gated channel alpha subunit 5* (*SCN5A*; OMIM *600163). To date, the employment of traditional approaches has limited detection rates for large genomic rearrangements resulting in CNVs. However, efforts have begun to evaluate the occurrence of CNVs in CNPs (Table 2).

In a study of 42 unrelated LQTS patients who tested negative for point mutations, two CNVs were identified in *KCNQ1* [38]. MLPA approach revealed a 5306 bp deletion (c.478-5001_604 + 178del) involving exon 3, that was predicted to generate a PTC, in a 10-year-old Caucasian boy. A second deletion in the *KCNQ1* gene was described in a 17-year-old Caucasian girl. It results in a complete deletion of exon 7 and skipping of exon 8 that was hypothesized to cause abnormal splicing [38]. Subsequently, another study detected CNVs in *KCNQ1* and *KCNH2* in 3% of 93 LQTS patients, who were negative for SNVs in these genes, reporting three deletions in total [39]. According to the authors, these findings support the inclusion of CNV screening in the genetic evaluation of LQTS, particularly in *KCNQ1* and *KCNH2*, as the CNV detection rate in these genes appears to be higher than SNVs in other LQT-related genes [39].

CNVs in potassium channel genes were also identified in 4.7% of a cohort of 127 LQTS patients, with deletions in *KCNQ1*, *KCNH2*, and *KCNE1*, some of which were classified as affecting function (AF) or probably affecting function (PAF) [32].

Recently, WGS showed its contribution in identifying CNVs missed by WES [40]. Specifically, three pathogenic variants were identified by WGS in a cohort of 25 WES-negative patients with suspected CNPs [40].

Another study described the role of CNVs in patients with CNPs, summarizing data extrapolated from the literature (119 patients) and their laboratory data (21 patients), all of whom tested positive for CNVs, yielding an overall detection rate of 5% [41]. The most frequently affected genes in LQTS were *KCNQ1* and *KCNH2*, with 35 and 25 CNVs reported, respectively. Additionally, CNVs in *SCN5A* were associated with BrS in patients, predominantly males, who had experienced major cardiac events. Specifically, deletions of exon 3 in three male family members and deletions of exons 15–16 in two unrelated families were described [41].

Finally, a MLPA-based study, involving 140 BrS probands, reported four different *SCN5A* CNVs (three deletions and one duplication) leading to phenotypes similar to those caused by nonsense or missense mutations. Importantly, these rearrangements were found to result in over 90% reduction in peak INa, functionally underscoring their pathogenic relevance and the importance of incorporating CNV screening into routine diagnostics [42]. Also, a large deletion of exon 23 in the *SCN5A* gene was identified in a patient with a BrS type 1 pattern who underwent genetic testing after three episodes of syncope at rest [43]. The authors hypothesized that transcripts produced by the truncated allele are subject to nonsense-mediated RNA decay, leading to a condition of haploinsufficiency for *SCN5A*. Cascade testing confirmed the same deletion in the patient’s father and two sisters, facilitating targeted family surveillance [43].

Regarding CPVT, there is emerging evidence suggesting a potential involvement of CNVs. Notably, one study identified a possible homozygous duplication encompassing the entire *TECRL* gene (*TECRL* OMIM #617242) and the first exon of *EPH Receptor A5* (*EPHA5* OMIM #600004). CNV detection was performed using NGS and subsequently confirmed by qRT-PCR. However, due to the inability to conduct familial segregation analysis, the pathogenicity of the identified variant could not be definitively established. Nonetheless, authors suggest that the duplication could lead to altered expression of *Trans-2,3-Enoyl-CoA Reductase-Like*, plausibly contributing to the disease, given its previously reported association with CPVT [44].

Similarly, a 1.1 kb deletion of exon 3 in *RYR2* was identified in two families with complex phenotypes consisting of CPVT combined with atrioventricular (AV) block, sinoatrial node (SAN) dysfunction, atrial fibrillation (AF), and atrial standstill. The large in-frame deletion in the N-terminal region of *RYR2* was segregated in 16 affected relatives of the two unlinked families, thus explaining such a complex phenotype [45]. The same deletion was identified in multiple CPVT-affected individuals within the same family. In this case, prior functional studies demonstrated that this specific deletion impairs *RYR2* function, thereby supporting its pathogenic role in conjunction with the observed clinical phenotype [46]. In the same genomic context of *RYR2* exon 3, another deletion of 698 bp has been identified. The affected individual showed severe CPVT phenotype and sudden cardiac arrest (SCA), and the development of LVNC concurrently with worsening arrhythmias led to the interpretation of a disease-causing deletion [47].

These studies show a non-negligible proportion of patients with genetically elusive CNPs, without extensive genomic rearrangements involving canonical susceptibility genes.

Although current cohorts remain limited in size, expanding genetic testing protocols to include CNV analysis appears critical for improving diagnostic yield and guiding clinical management in patients with inherited CNPs.

## 4. Discussion

The diagnosis of genetic CMPs and CNPs remains particularly challenging, primarily due to the significant genetic and clinical heterogeneity underlying these conditions.

Numerous studies and clinical screenings have consistently reported a substantial portion of patients with no identifiable genetic cause, despite being clinically affected. Importantly, such proportion often exceeds 50% of cases [21].

This considerable diagnostic gap has highlighted the necessity of expanding genetic analyses to explore not only novel causative factors but also alternative classes of genomic variants, including the CNVs.

The present review highlighted the identification of pathogenic CNVs, especially deletions, in patients who had previously tested negative via standard analyses.

Nevertheless, diagnostic yields vary considerably across studies. Some investigations have demonstrated limited success in identifying CNVs.

Nonetheless, a key observation emerging from our literature review is that most CNVs identified to date are located within genes already established as causative for CMPs and CNPs [13]. In particular, this aspect emerges from a large-scale study utilizing genome-wide approaches in the 100 K Genomes Project cohort. The research revealed that CNVs accounted for approximately 5% of 111 pediatric cardiomyopathy cases and included deletions in *TTN* and *FLNC* genes among patients with DCM [48]. These findings support the inclusion of CNV analysis in routine genetic diagnostics, particularly for coding and regulatory regions of genes listed in current clinical guidelines. To the best of our knowledge, a standardized workflow for integrating CNV analysis into the diagnostic routine for CMPs and CNPs has not yet been clearly established.

From a diagnostic prospective, we believe CNVs should be systematically evaluated in these clinically relevant genes to optimize genetic resolution by means of targeted solutions. In particular, incorporating the analysis of CNVs involving recommended genes for CMPs and CNPs, namely *MYBPC3* (for HCM) or *PKP2*, *DSC2*, and *DMD* (for ACM and DCM) or *SCN5A*, *KCNQ1*, and *RYR2* (for CNPs), into routine diagnostic workflows could aid in resolving unsolved cases or expedite the referral toward more in-depth analyses, such as WES or WGS. To this end, the pathogenic impact of CNVs should be clarified through familial co-segregation analyses or validated by collecting experimental evidence. On this subject, functional tests can directly assess the impact of CNVs on gene expression and protein function through targeted or large-scale approaches, such as reporter gene analysis and multiplex assays of variant effect (MAVEs). However, the application of these technologies can be both time-consuming and costly, and their implementation is often challenging, making it difficult to elucidate the functional consequences of CNVs [49], as further highlighted by the present review, which indicates that the majority of the studies discussed did not include a functional assessment of the identified CNVs. Therefore, familial segregation analyses remain crucial for supporting the interpretation of CNVs pathogenicity.

In addition, such diagnostic workflow that primarily focuses on the detection of CNVs in recommended genes and extends to genome-wide analysis only in the case of negative results may also be appropriate in complex cases. This is particularly relevant given the phenotypic heterogeneity arising from the coexistence of CMPs and CNPs [50], as corroborated by the abovementioned CPVT/LVNC case harboring a *RYR2* deletion [47].

The present review also highlighted that CNVs were investigated with different techniques, such as MLPA, array comparative genomic hybridization (aCGH), and SNP arrays. Notably, CNVs can also be identified through high-coverage WES data, which provides a feasible strategy to detect such variants across the coding regions.

Importantly, a significant limitation of CNVs analysis lies in the technological constraints of standard short-read sequencing, which is not optimized for accurately detecting complex structural changes. In this regard, long-read genome sequencing technologies have emerged as powerful tools, offering enhanced resolution of CNVs across the genome [51,52,53].

The advent of more refined sequencing platforms will also enhance the research, namely the exploration of CNVs in novel or less characterized genes or even in poorly known regulatory regions. Such exploratory analysis may help identify new candidate genes involved in disease pathogenesis through mechanisms such as loss of function or haploinsufficiency, potentially expanding our understanding of the molecular underpinnings of these disorders.

Additionally, the exploration of CNVs in non-coding or intergenic regions, which may harbor regulatory elements or influence higher-order chromatin architecture, has the potential to elucidate novel disease mechanisms, for example, disruptions in chromosomal organization have been implicated in DCM pathogenesis [54]. Importantly, the implementation of advanced computational frameworks based on Artificial Intelligence (AI) has the potential to assist specialists in assessing the clinical relevance of CNVs. These tools could enhance classification accuracy, especially if they are designed to learn from prior CNV interpretations, experimental validation data, and clinical information, as mentioned above, while maintaining explainability to allow experts to critically evaluate and refine the results [55,56]. In conclusion, the comprehensive analysis of structural variants holds significant promise in enhancing the diagnostic yield and clinical management of inherited CMPs and CNPs. Integrating these analyses, both in guideline-recommended genes for diagnostic purposes and more broadly in exploratory research, will be instrumental in advancing precision medicine for these diseases.

## Figures and Tables

**Table 1:** Summary of the CNVs identified by the illustrated articles for each cardiomyopathy. The specific phenotype, type of CNV, and the associated gene, with related affected exons, are reported. The classification was reported when performed by the related study. N.A: not available.

Phenotype	Gene	CNV	Pathogenicity	Exon(s)	Reference
HCM	*MYBPC3*	Deletion	N.A.	Intron 32	[14]
	*MYBPC3*	Deletion	N.A.	Exons 27–35	[15,16]
	*MYBPC3*	Deletion	Likely Pathogenic	Exon 21	[17]
	*MYBPC3*	Deletion	Likely Pathogenic	Exons 4–5	
	*MYBPC3*	Deletion	Likely Pathogenic	Exons 1–5	
	*MYBPC3*	Deletion	Likely Pathogenic	Exons 4–7	
	*MYBPC3*	Deletion	Likely Pathogenic	All exons	
	*MYBPC3*	Deletion	Likely Pathogenic	Exons 1–17	
	*MYBPC3*	Deletion	Likely Pathogenic	Exon 18	
	*MYBPC3*	Deletion	Pathogenic	Exons 23–26	
	*MYBPC3*	Del/Ins	Likely Pathogenic	Exon 27	
	*MYH7*	Deletion	Pathogenic	All exons	
	*FHL1*	Deletion	Likely Pathogenic	Exons 1–7	
	*FHOD3*	Del/Ins	Pathogenic	Exon 15	
	*FHOD3*	Deletion	Pathogenic	Exons 15–16	
	*FHOD3*	Del/Ins	Pathogenic	Exons 15–16	
	*LAMP2*	Deletion	Likely Pathogenic	Exon 7	
	*PKP2*	Deletion	Likely Pathogenic	Exons 13–14	
	*PKP2*	Deletion	Pathogenic	Exon 8	
	*PLN*	Deletion	Pathogenic	Exons 1–2	
ACM	*PKP2*	Deletion	N.A.	Exons 1–14	[18]
	*PKP2*	Deletion	N.A.	Exons 2–14	
	*PKP2*	Deletion	N.A.	All exons	[19]
	*PKP2*	Deletion	N.A.	Exon 4	
	*PKP2*	Deletion	N.A.	Exons 6–11	
	*PKP2*	Duplication	N.A.	5′UTR-Exon1	
	*DSC2*	Duplication	N.A.	Exons 7–9	
	*DSC2/DSG2*	Deletion	N.A.	*DSC2* All exons/*DSG2* All exons	
	*PKP2*	Deletion	N.A.	Exons 1–14	[20]
	*PKP2*	Deletion	N.A.	Exons 2–14	
	*PKP2*	Deletion	N.A.	Exons 1–7	
	*PKP2*	Deletion	N.A.	Exon 4	
	*PKP2*	Deletion	N.A.	Exons 6–14	[21]
	*PKP2*	Deletion	N.A.	Exons 6–14	
	*PKP2*	Deletion	N.A.	Exon 12	
	*DMD*	Deletion	Likely Pathogenic	Exons 25–30	[17]
	*DMD*	Duplication	Likely Pathogenic	Exons 40–55	
	*DSP*	Deletion	Pathogenic	Exons 21–24	
	*FLNC*	Deletion	Likely Pathogenic	All exons	
	*FLNC*	Deletion	Pathogenic	Exons 3–48	
	*PKP2*	Deletion	Likely Pathogenic	Exon 8	
	*PKP2*	Deletion	Likely Pathogenic	Exon 4	
	*PKP2*	Deletion	Likely Pathogenic	Exon 10	
	*PKP2*	Duplication	Likely Pathogenic	Exons 8–10	
	*PKP2*	Del/Ins	Likely Pathogenic	Exons 5–14	
	*PKP2*	Deletion	Likely Pathogenic	Exons 13–14	
	*PKP2*	Deletion	Likely Pathogenic	Exons 13–14	
DCM	*BAG3*	Deletion	N.A.	Exon 4	[22]
	*LMNA*	Deletion	N.A.	Exons 3–12	[23]
	*GATA4*	Deletion	N.A.	All exons	[24]
	*DMD*	Deletion	N.A.	Exons 48–51	
	*DMD*	Duplication	N.A.	Exons 19–37	
	*LMNA*	Del/Ins	N.A.	NA	
	*PKP2*	Deletion	N.A.	Exon 8	
	*DSP*	Deletion	N.A.	Exon 1	
	*RBM20*	Deletion	N.A.	NA	[25]
	*TTN*	Deletion	Pathogenic	Exons 224–335	[26]

**Table 2:** Summary of the CNVs identified by the illustrated articles for CNPs. The specific phenotype, type of CNV, and the associated gene with related affected exons are reported. The classification was reported when performed by the related study. N.A.: not available.

Phenotype	Gene	CNV	Pathogenicity	Exon(s)	Reference
LQTs	*KCNQ1*	Deletion	N.A.	Exon 3	[38]
LQTs	*KCNQ1*	Deletion	N.A.	Exon 7	
LQTs	*KCNQ1*	Deletion	N.A.	Exons 7–8	[39]
LQTs	*KCNH2*	Deletion	N.A.	Exons 4–15	
LQTs	*KCNH2*	Deletion	N.A.	Exons 1–15	
LQTs	*KCNQ1*	Deletion	Pathogenic	Exons 8–9	[32]
LQTs	*KCNH2*	Deletion	Pathogenic	Exons 1–15	
LQTs	*KCNE1*	Deletion	Pathogenic	Exon 3	
LQTs	*KCNQ1*	Deletion	Pathogenic	Exon 2	[40]
LQTs	*KCNQ1*	Deletion	N.A.	Exon 1	[41]
LQTs	*KCNQ1*	Deletion	N.A.	Exon 7	
LQTs	*KCNQ1*	Deletion	N.A.	Exons 9–10	
LQTs	*KCNQ1*	Deletion	N.A.	Exon 16	
BrS	*SCN5A*	Deletion	N.A.	Exon 3	
BrS	*SCN5A*	Deletion	N.A.	Exons 15–16	
BrS	*SCN5A*	Deletion	N.A.	All exons	[42]
BrS	*SCN5A*	Deletion	N.A.	Exon 4	
BrS	*SCN5A*	Deletion	N.A.	Exon 24	
BrS	*SCN5A*	Duplication	N.A.	Exons 17–24	
BrS	*SCN5A*	Deletion	N.A.	Exon 23	[43]
CPVT	*TECRL/EPHA5*	Duplication	Variant of Uncertain Significance	Exons 1–12/Exon 1	[44]
CPVT	*RYR2*	Deletion	N.A.	Exon 3	[45]
CPVT	*RYR2*	Deletion	Pathogenic	Exon 3	[46]
CPVT	*RYR2*	Deletion	Pathogenic	Exon 3	[47]

## Data Availability

Not applicable.

## Abbreviations

The following abbreviations are used in this manuscript:

SCD	Sudden Cardiac Death
CMPs	Cardiomyopathies
DCM	Dilated Cardiomyopathy
HCM	Hypertrophic Cardiomyopathy
ACM	Arrhythmogenic Cardiomyopathy
CNPs	Channelopathies
LQTS	Long QT Syndrome
BrS	Brugada Syndrome
CPVT	Catecholaminergic Polymorphic Ventricular Tachycardia
SVs	Structural Variants
CNVs	Copy Number Variants
SNVs	Single Nucleotide Variants
Indels	Insertions/Deletions
MLPA	Multiplex Ligation-dependent Probe Amplification
PTCs	Premature Termination Codons
AF	Affecting Function
PAF	Probably Affecting Function
aCGH	array Comparative Genomic Hybridization
ACMG	American College of Medical Genetics and Genomics
AI	Artificial Intelligence
